# 101. PCV13 Pediatric Vaccination Disparity and Impact Due to COVID-19 Pandemic in the US

**DOI:** 10.1093/ofid/ofab466.101

**Published:** 2021-12-04

**Authors:** Liping Huang, Jennifer L Nguyen, Johnna Perdrizet, Tamuno Alfred, Adriano Arguedas

**Affiliations:** 1 Pfizer, Inc., Collegeville, PA; 2 Pfizer Inc., New York, New York; 3 Pfizer Inc, Collegeville, Pennsylvania

## Abstract

**Background:**

Existing disparities in vaccination rates across different social and demographic groups in the US may have been exacerbated during the Coronavirus Disease 2019 (COVID) pandemic, leaving some children at risk for vaccine-preventable diseases. This study examined sociodemographic and risk factors of PCV13 infant primary series vaccination completion, before and during COVID.

**Methods:**

Retrospective data from the Optum’s de-identified Clinformatics Data Mart Database were used to create 3 cohorts: C1, Pre-COVID; C2, During COVID; C3, Cross-COVID (Figure 1). C1 and C3 (C1&3) were combined and compared with C2 for primary dosing completion before and during COVID according to infant/caregiver characteristics. Full completion (FC) was defined as receipt of 3 doses of PCV13 within 8 months of birth. Multivariable logistic regression was used to compare FC vs. partial completion or no vaccine. Descriptive analyses were used to compare FC before and during COVID within subgroups.

Figure 1: Study population and inclusion criteria

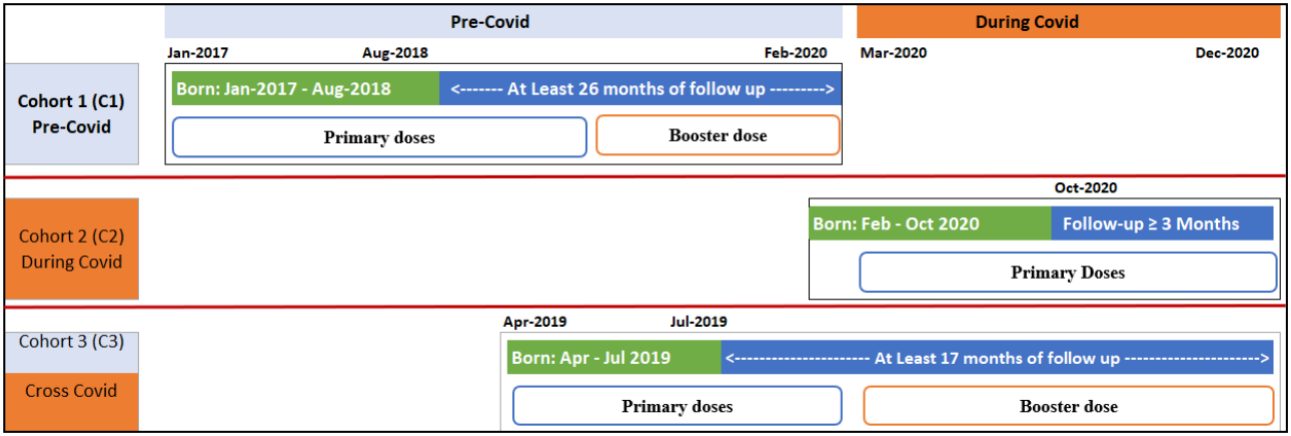

**Results:**

A total of 132,183 and 16,522 infants with at least 8 months of follow up time were enrolled in C1&3 and C2, respectively. FC was significantly higher before COVID-19 (adjusted odds ratio = 1.12, 95% CI: 1.07-1.17). Adjusting for COVID, FC was significantly lower in infants who were Black, with co-morbidities or risk factors, living in households with >1 children or no children, household annual income < &99k, residing in a neighborhood with median education of high school or below, and whose primary caregiver was aged <25 years (Table 1). Comparing FC before and during COVID, the % decline relative to pre-COVID was > 2% among infants who were White, residing in the Mountain, New England or Pacific regions, in a household with 2 children, >&100k annual income, employer-based insurance or HMO, and median neighborhood education of bachelor degree plus (Table 2).

Table 1. Multivariable binomial logistic regression results for PCV13 full primary dosing completion vs. not full completion (partial or no vaccine), N=144,799*

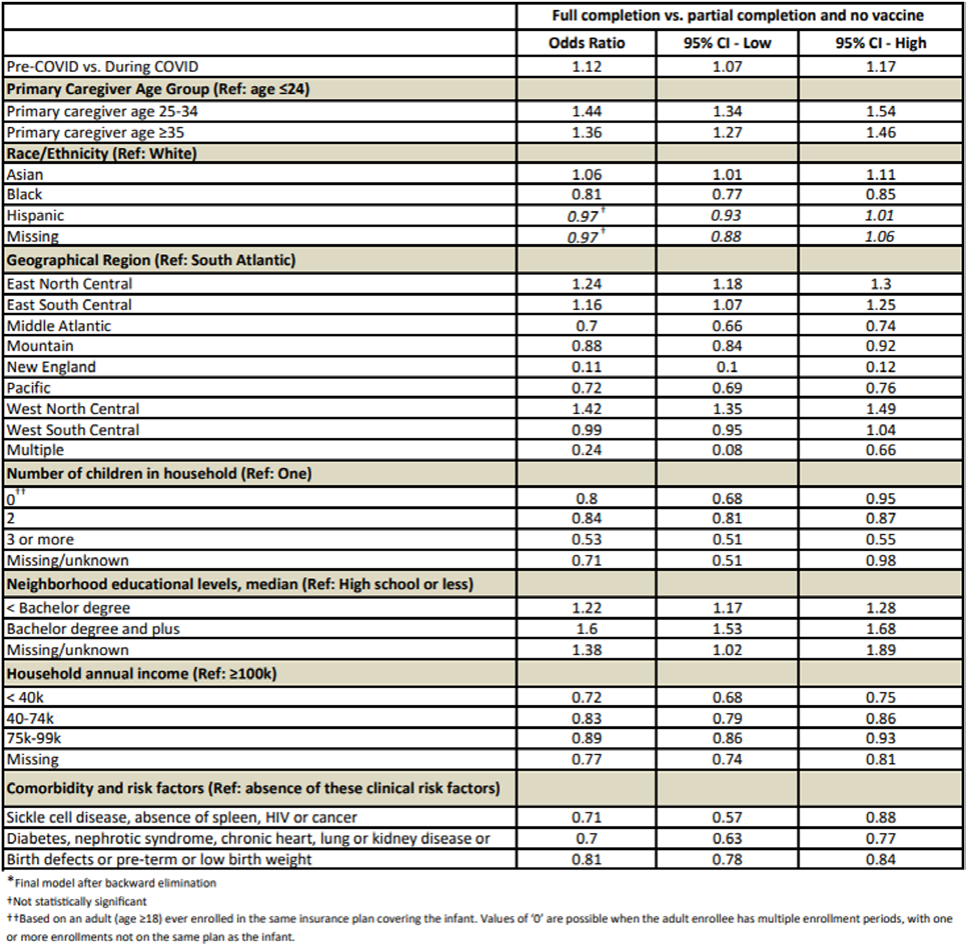

Table 2. Primary dosing full completion rate pre-COVID vs. during COVID by social, demographic, and clinical risk factors

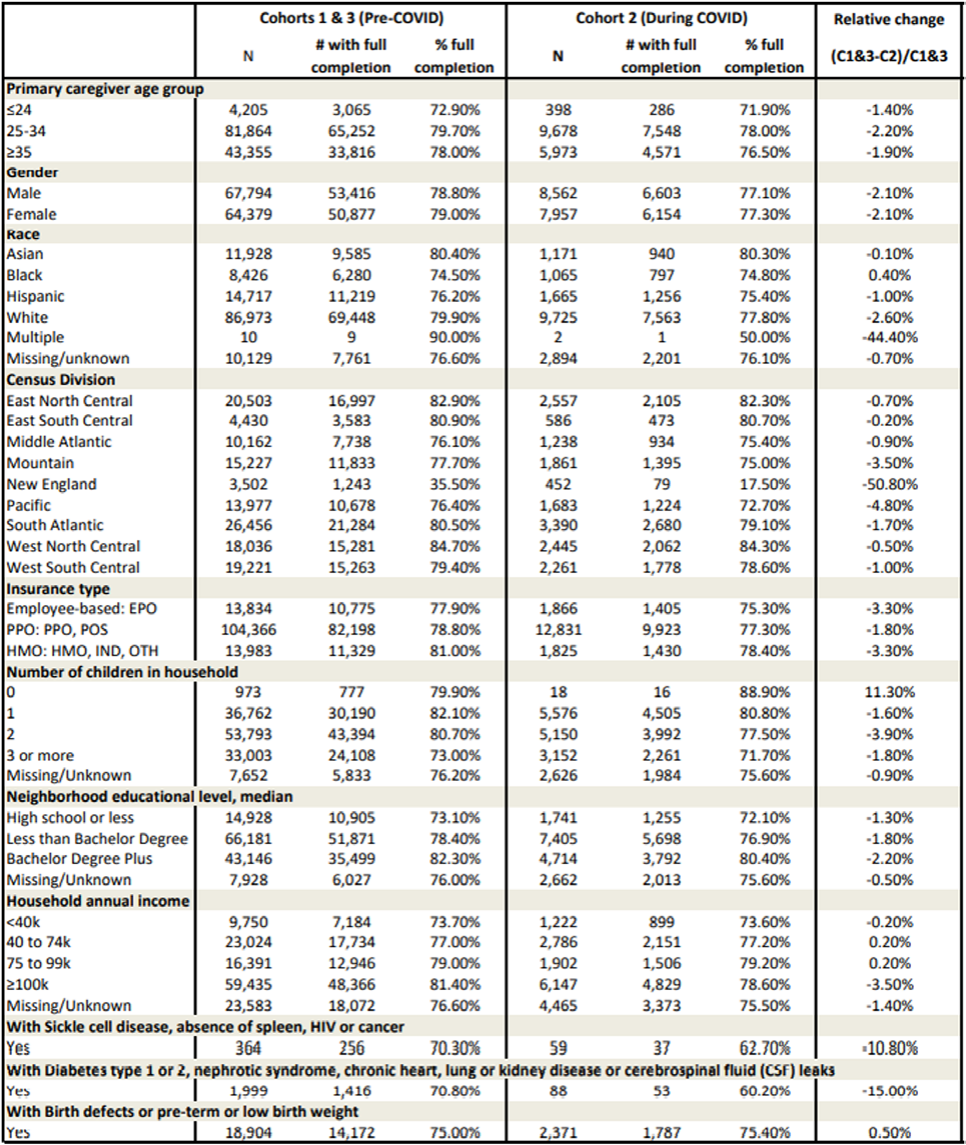

**Conclusion:**

Health inequities in PCV13 primary series completion existed prior to COVID-19 and have remained during the pandemic. Our results, however, suggest that during the pandemic, groups traditionally considered to have better healthcare access (Whites, higher income, more education) had more impact on vaccine uptake. Further research is needed to confirm these trends as COVID mitigation measures subside.

**Disclosures:**

**Liping Huang, MD, MA, MS**, **Pfizer Inc** (Employee) **Jennifer L Nguyen, ScD, MPH**, **Pfizer Inc.** (Employee) **Johnna Perdrizet, MPH**, **Pfizer Inc** (Employee) **Tamuno Alfred, PhD**, **Pfizer Inc.** (Employee) **Adriano Arguedas, MD**, **Pfizer** (Employee)

